# Characterisation of atypical enteropathogenic *E. coli *strains of clinical origin

**DOI:** 10.1186/1471-2180-9-117

**Published:** 2009-06-03

**Authors:** Sharon M Tennant, Marija Tauschek, Kristy Azzopardi, Andrea Bigham, Vicki Bennett-Wood, Elizabeth L Hartland, Weihong Qi, Thomas S Whittam, Roy M Robins-Browne

**Affiliations:** 1 Department of Microbiology and Immunology, The University of Melbourne, and Murdoch Childrens Research Institute, Royal Children's Hospital, Victoria 3010, Australia; 2 Microbial Evolution Laboratory, National Food Safety and Toxicology Center, Michigan State University, Michigan, USA; 3 Center for Vaccine Development, University of Maryland School of Medicine, Baltimore, MD 21201, USA; 4 Functional Genomics Center Zurich, Uni/ETH Zurich, Winterthurerstrasse 190/Y32 H66, CH-8057 Zurich, Switzerland

## Abstract

**Background:**

Enteropathogenic *E. coli *(EPEC) is a prominent cause of diarrhoea, and is characterised in part by its carriage of a pathogenicity island: the locus for enterocyte effacement (LEE). EPEC is divided into two subtypes according to the presence of bundle-forming pili (BFP), a fimbrial adhesin that is a virulence determinant of typical EPEC (tEPEC), but is absent from atypical EPEC (aEPEC). Because aEPEC lack BFP, their virulence has been questioned, as they may represent LEE-positive Shiga toxin-producing *E. coli *(STEC) that have lost the toxin-encoding prophage, or tEPEC that have lost the genes for BFP. To determine if aEPEC isolated from humans in Australia or New Zealand fall into either of these categories, we undertook phylogenetic analysis of 75 aEPEC strains, and compared them with reference strains of EPEC and STEC. We also used PCR and DNA hybridisation to determine if aEPEC carry virulence determinants that could compensate for their lack of BFP.

**Results:**

The results showed that aEPEC are highly heterogeneous. Multilocus sequence typing revealed that 61 of 75 aEPEC strains did not belong to known tEPEC or STEC clades, and of those that did, none expressed an O:H serotype that is frequent in tEPEC or STEC strains associated with disease. PCR for each of 18 known virulence-associated determinants of *E. coli *was positive in less than 15% of strains, apart from NleB which was detected in 30%. Type I fimbriae were expressed by all aEPEC strains, and 12 strains hybridised with DNA probes prepared from either *bfpA *or *bfpB *despite being negative in the PCR for *bfpA*.

**Conclusion:**

Our findings indicate that clinical isolates of aEPEC obtained from patients in Australia or New Zealand are not derived from tEPEC or STEC, and suggest that functional equivalents of BFP and possibly type I fimbriae may contribute to the virulence of some aEPEC strains.

## Background

Strains of enteropathogenic *E. coli *(EPEC) are a well-recognised cause of diarrhoea, particularly in children in less developed countries [1,2]. EPEC are characterised in part by their ability to induce attaching-effacing (A/E) lesions in the intestine [3-5]. These lesions are comprised of bacteria intimately attached to the intestinal mucosa at sites of cytoskeletal rearrangements leading to characteristic morphological changes, known as cupping and pedestal formation, accompanied by the absence of microvilli. The genes required for the production of these lesions are located on a pathogenicity island known as the locus for enterocyte effacement (LEE), which encodes (i) intimin, an outer membrane protein product of the *eae *gene that acts as an adhesin, (ii) a type III protein secretory system, and (iii) several effector proteins secreted by the type III system, including a translocated intimin receptor, Tir, which, once bound to intimin, serves as an anchor for host cytoskeletal proteins [6]. EPEC is divided into two subtypes: typical and atypical. Typical EPEC (tEPEC) strains carry a ca. 90-kb EPEC adherence factor plasmid (pEAF) that encodes type IV-like bundle-forming pili (BFP) [7]. The latter facilitate the adherence of bacteria to the intestinal mucosa and to each other, allowing them to form micro-colonies on epithelial cells in vitro and in vivo [8,9]. Studies with adult volunteers have demonstrated that intimin, pEAF and BFP are essential virulence determinants of EPEC [10-12]. Interestingly, there is evidence that a subset of EPEC strains, known as atypical EPEC (aEPEC), which lack pEAF and BFP, are also pathogenic [2].

aEPEC is defined as *E. coli *which possess LEE, but lack pEAF/BFP and do not produce Shiga toxins [13]. Evidence of the pathogenicity of aEPEC comes from case control studies of paediatric diarrhoea in several countries throughout the world, including Australia, Iran, Norway, Peru, Poland, South Africa, the United Kingdom and the USA (reviewed in [2,14]). In addition, at least three separate studies have shown an association between infection with aEPEC and persistent diarrhoea in children [14-16]. Notwithstanding these reports, the pathogenicity of aEPEC remains controversial, chiefly because several studies have found aEPEC in patients with diarrhoea and control subjects at similar frequencies. These conflicting observations prompt the question of whether aEPEC comprise a homogeneous group of pathogens with shared virulence determinants, such as adhesins analogous to BFP, or whether they are heterogeneous, with one or more subsets being more virulent than others. Although some clinical isolates of A/E strains of *E. coli *which meet the definition of aEPEC, appear to be Shiga-toxin producing strains of *E. coli *(STEC) that have lost the Shiga toxin-encoding bacteriophage(s) during passage through the intestine [17], others may be tEPEC strains that have lost pEAF [12]. Alternatively, aEPEC may represent a distinct subset of human-specific strains of A/E *E. coli *or be acquired from domestic animals, such as calves and rabbits, that are commonly infected with EPEC strains, which lack pEAF [18,19]. In this study we characterised a large number of clinical isolates of aEPEC from humans by multi-locus sequence typing (MLST), PCR and/or DNA hybridisation for *E. coli *virulence-associated determinants, intimin type, HEp-2 adherence pattern and type 1 pilus production as a way of addressing these questions.

## Results

### MLST analysis

**Figure 1:**
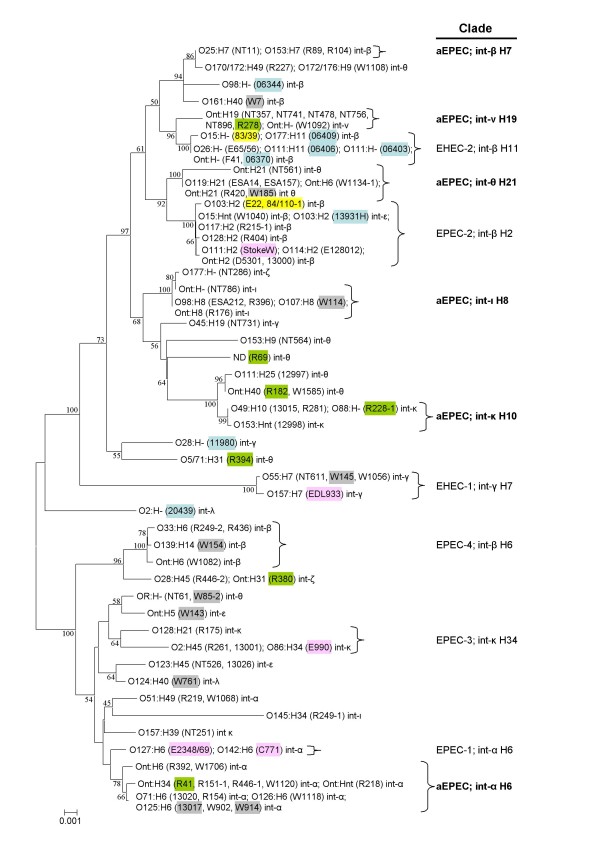
**Phylogenetic relationships of sequence types of 95 strains of attaching-effacing *E. coli***. An unrooted phylogenetic tree was constructed by the neighbour-joining algorithm based on the Kimura two-parameter model of nucleotide substitution. Bootstrap values greater than 50% based on 500 replications are given at the internal nodes. Strain names highlighted in pink are reference strains of typical EPEC or STEC; those highlighted in pale blue and yellow were originally isolated from cattle and rabbits, respectively, those highlighted in green were from children with persistent diarrhoea, and those highlighted in grey were from humans without diarrhoea. The right hand column indicates distinctive aEPEC clades in boldface type. The intimin and flagella type is shown for each clade. Abbreviations: nt, non-typable; int, intimin; ND, not determined.

We have previously reported that aEPEC isolates obtained during a water quality study were heterogeneous in terms of serotype, intimin type and patterns of adherence to HEp-2 cells [20]. This overall heterogeneity was confirmed by MLST analysis, which showed that 56 of the 79 aEPEC strains of human origin investigated in the study belonged to one of 11 different clades and that 23 strains could not allocated to a clade (Figure 1). As observed with phylogenetic analyses of A/E strains of *E. coli *in general, there was a tendency for each clade to contain strains with the same intimin and flagellar type. Five of 11 clades which contained aEPEC strains in this study were clades that include either tEPEC or STEC strains, whereas six clades were apparently distinct for aEPEC. These six clades comprised one which contained three strains with intimin-β and H7 (and O-antigens, O25 or O153); one clade with seven strains with intimin-ν and H19 (all O-nontypable [nt]); one clade with six strains with intimin-θ and H21 (O119 and Ont); a clade with five strains with intimin-ι and H8 or H- (O98, O107 or O177); one with four strains with intimin-κ and H10 or H- (O49, O88, and O153), and one with 13 strains with intimin-α and H6 or H34 (O71, O125, O126, and Ont). The last-mentioned clade was closely related to a tEPEC clade (EPEC-1), which also comprises strains with intimin-α and flagellar antigen, H6.

Of the strains that clustered with known clades of tEPEC or EHEC, three (all intimin-γ and O55:H7) belonged to the EHEC-1 clade, which also includes the pandemic, prototypical O157:H7 EHEC clone. Five aEPEC isolates grouped within the EPEC-2 clade which includes pEAF/BFP-positive strains with intimin-β and H2. The aEPEC serotypes in this clade included O15:Hnt, O114:H2, O117:H2, O128:H2, and Ont:H2. This clade also contained the prototypical aEPEC strain, E128012 [12], two rabbit-specific EPEC (REPEC) strains, 84/110/1 and E22 (both of which carry intimin-β and are serotype O103:H2), and a calf isolate, also O103:H2, but with intimin-ε. Four aEPEC strains were assigned to the EPEC-4 clade (intimin-β and H6). All of these strains were intimin-β; two were O33:H6, and the others were O139:H14 and Ont:H6. Two aEPEC strains, both O2:H4, intimin-κ, belonged to the EPEC-3 clade, which also included tEPEC strain O86:H34, intimin-κ. Overall, three of 75 Australian and New Zealand isolates from humans reported here belonged to EHEC clades, 11 fell within EPEC clades, 38 belonged to clades distinct for aEPEC, and 23 could not be classified.

Of the eight Australian calf isolates examined, three were not assigned to a particular clade, and four belonged to the EHEC-2 clade (intimin-β and H11), but were of different serotypes from each other (O111:H-, O111:H11, O177:H11 and Ont:H-). This clade also included archetypal aEPEC strains E65/56 (O26:H-) and F41 (Ont:H-), which were isolated in Europe more than 50 years ago, and a well-studied, REPEC strain, 83/39 (O15:H-), in which Ral, a K88-like adhesin, was first identified [21]. None of the Australian or New Zealand strains of human origin investigated in this study belonged to this clade, suggesting that there was no major exchange of aEPEC strains between the cattle and humans from whom these bacteria were obtained. One calf isolate (O103:H2) was assigned to the EPEC-2 clade, which also contained REPEC strains E22 and 84/110-1 (also O103:H2), tEPEC strain, Stoke W (O111:H2), and the prototypical aEPEC strain, E128012 (O114:H2). No aEPEC strains carrying intimin-β2, -ξ, -o, -ρ or -σ were identified in the entire collection of 87 test and 8 reference strains reported here.

### Frequency of adhesins and other virulence determinants of pathogenic *E. coli *in aEPEC strains

None of the 67 Australian aEPEC strains of human origin investigated in this study was positive in the PCR for genes encoding the following adhesins of pathogenic *E. coli*: BFP, Lda, Pap, Saa, Afa, Sfa/Foc, K88, K99, Af/R2 or RalG (Table 1). On the other hand, all strains were positive in the PCR for FimH of Type 1 pili. Moreover, all isolates exhibited mannose-sensitive haemagglutination, indicating that they produced functional Type 1 pili.

**Table 1:** Characteristics of atypical EPEC strains that were positive in one or more PCR or DNA hybridization assays for virulence-associated determinants of *E. coli*^a^

				Result of assay using:										
					
				PCR for								Probe for		
						
Strain	Serotype	Clade	Intimin	Afa	Afr/I	AggA	Cdt	Efa1	Iha	LpfD	NleB	BfpA	BfpB	HEp-2 adhesion^b^
ESA14	O119:H21	aEPEC3	θ	-	-	-	-	-	-	-	+	-	-	NA
ESA157	O119:H21	aEPEC3	θ	-	-	-	-	-	-	-	+	-	-	NA
ESA212	O98:H8	aEPEC4	ι	-	-	-	-	-	-	-	-	+	-	NA
NT11	O25:H7	aEPEC1	β	-	-	-	-	+	-	+	+	-	-	IA
NT286	O177:H-		ζ	-	-	-	-	-	+	-	-	-	-	NA
NT357	Ont:H19	aEPEC2	ν	-	-	-	-	-	-	+	-	-	-	IA
NT478	Ont:H19	aEPEC2	ν	-	-	-	-	-	-	+	-	-	-	NA
NT526	O123:H45		ε	-	-	-	+	-	-	-	-	-	-	IA
NT561	Ont:H21	aEPEC3	θ	-	-	-	-	-	-	-	+	-	-	AA
NT611	O55:H7	EHEC1	γ	-	-	-	-	+	-	-	+	-	-	AA
NT731	O45:H19		γ	+	-	-	-	-	+	-	-	-	-	NA
NT741	Ont:H19	aEPEC2	ν	-	-	-	-	-	-	+	-	-	-	IA
NT756	Ont:H19	aEPEC2	ν	-	-	-	-	-	-	+	-	-	-	IA
NT786	OR:H-	aEPEC4	ι	-	-	-	-	-	+	-	-	-	-	AA
NT896	Ont:H19	aEPEC2	ν	-	-	-	-	-	-	+	-	-	-	IA
R104	O153:H7	aEPEC1	β	-	+	-	-	+	-	+	+	-	-	AA
R151-1	Ont:H34	aEPEC6	α	-	-	-	+	-	-	-	-	-	+	AA
R176	Ont:H8	aEPEC4	ι	-	-	-	-	-	-	-	-	+	-	NA
R182	OR:H40		θ	-	-	-	-	-	-	-	+	+	-	IA
R215-1	O117:H2	EPEC2	β	-	-	-	-	+	-	+	+	-	-	IA
R218	Ont:R	aEPEC6	α	-	-	-	+	-	-	-	-	-	+	AA
R219	O51:H49		α	-	-	+	-	-	-	-	-	-	-	AA
R227	O170/172:H49		θ	-	-	-	-	-	-	-	+	-	-	IA
R228-1	O88:H-	aEPEC5	κ	-	-	-	-	-	-	-	-	+	-	NA
R249-1	O145:H34		ι	-	-	-	-	-	-	-	-	-	+	AA
R278	Ont:H19	aEPEC2	ν	-	-	-	-	-	-	+	-	-	-	IA
R281	O49:H10	aEPEC5	κ	-	-	-	-	-	-	-	-	+	-	NA
R380	Ont:H31		ζ	-	-	-	+	-	-	-	-	-	-	AA
R392	Ont:H6	aEPEC6	α	-	-	-	-	-	-	-	-	-	+	AA
R394	O5/71:H31		θ	-	-	-	-	-	-	-	+	-	-	IA
R404	O128:H2	EPEC2	β	-	-	-	+	-	-	+	-	-	+	LA-L
R420	Ont:H21	aEPEC3	θ	-	-	-	-	-	-	-	+	-	-	IA
R446-1	Ont:H34	aEPEC6	α	-	-	-	+	-	-	-	-	-	+	AA
R446-2	O28:H45		ζ	-	-	-	+	-	-	-	-	-	-	AA
R69	NT		θ	-	-	-	-	-	-	-	+	-	-	IA
R89	O153:H7	aEPEC1	β	-	+	-	-	+	-	+	+	-	-	IA
W1040	O15:Hnt	EPEC2	β	-	-	-	-	+	-	+	+	-	-	LA-L
W1056	O55:H7	EHEC1	γ	-	-	-	-	+	-	-	+	-	-	IA
W1092	OR:H-	aEPEC2	ν	-	-	-	-	-	-	+	-	-	-	IA
W1108	O176/172:H49		θ	-	-	-	-	-	-	-	+	-	-	IA
W1134-1	Ont:H6	aEPEC3	θ	-	-	-	-	-	-	-	+	-	-	NA
W114	O107:H8	aEPEC4	ι	-	-	-	-	-	-	-	-	+	-	NA
W145	O55:H7	EHEC1	γ	-	-	-	-	+	-	-	+	-	-	AA
W1585	Ont:H40		θ	-	-	-	-	-	-	-	+	-	-	NA
W185	Ont:H21	aEPEC3	θ	-	-	-	-	-	-	-	-	-	-	IA

^a ^No strain was positive in the PCR for BFP, Lda, Pap, Saa, Afa, Sfa/Foc, K88, K99, Af/R2 or RalG, and no strain hybridised with a probe for K88. All strains were positive in the PCR for FimH. ^b ^Adherence pattern after 3 hours' incubation with HEp-2 cells: AA, aggregative adherence; IA, indeterminate pattern; LA-L, localised-like adherence; NA, non-adherent.

One strain, NT731 (O45:H19, intimin-γ) was the only one of the 67 aEPEC isolates investigated that was positive in the PCR for Afa, a virulence determinant of uropathogenic *E. coli *that also serves as an adhesin in some EPEC strains [22,23].

Two strains, R89 and R104, which belonged to a distinct aEPEC clade, characterised by intimin-β and H7 flagella, were positive in the PCR for AF/R1, a fimbrial adhesin and essential virulence determinant of the prototypical REPEC strain, RDEC-1 [24]. One strain, R219 (O51:H49, intimin-α), was the only one that tested positive for AggA, the pilin subunit of enteroaggregative *E. coli *[25]. Three strains, NT286, NT731, and NT786, were positive in the PCR for Iha, an adherence-conferring protein that is highly prevalent in STEC [26]. The Iha-positive aEPEC strains identified here were unrelated to each other and to STEC in terms of MLST type, intimin type and serotype (Table 1, Figure 1).

The PCR for long polar fimbriae (Lpf) was positive in 13 (19%) of 67 strains, all of which were from patients with diarrhoea. Lpf-positive strains occurred within the EPEC-2 clade (3 strains), the aEPEC clade with intimin-ν and H19 (7 strains), and the aEPEC clade with intimin-β and H7 (3 strains). Overall, seven of the LPF-positive isolates carried intimin-ν, and six had intimin-β.

We and others have previously reported that certain determinants of EPEC, which are not encoded by LEE or the EAF plasmid, such as Efa1, NleB and the cytolethal distending toxin (Cdt) are associated with virulence in attaching-effacing *E. coli *[15,27]. NleB was detected in 20 (30%) of the 67 strains tested, whereas Efa1 was detected in 8 strains (12%), all of which were also positive for NleB. NleB-positive strains were distributed amongst the following clades: EHEC-1 (3 strains), EPEC-2 (2 strains), aEPEC-1 (intimin-β, H7; 3 strains), aEPEC-3 (intimin-θ, H21 [or H6]; 6 strains). Six NleB-positive isolates could not be assigned to a clade, although all carried intimin-θ (Table 1). The Efa1-positive strains occurred within the EHEC-1 and EPEC-2 clades, as well as within the aEPEC-1 clade that was characterised by strains with intimin-β and H7. Seven (10%) strains were positive in the PCR for Cdt. Three of these strains belonged to the aEPEC-6 clade (intimin-α and H34), one belonged to EPEC-2 (intimin-β, H2), and three were unassigned (Table 1).

### DNA hybridization

To determine if aEPEC carry DNA sequences related to those that code for the production of BFP, but were not amplified by the PCR for BfpA, we investigated the aEPEC strains by DNA hybridisation using probes derived from the *bfpA *and *bfpB *genes of EPEC strain E2348/69. Unexpectedly, six isolates (ESA212, R176, R182, R228-1, R281, and W114) hybridised with the BfpA probe at high stringency. Three of these strains belonged to the aEPEC clade with intimin-ι and H8, but they belonged to different O-serogroups. The other three probe-positive strains also differed from each other. Six strains hybridised with the BfpB probe. Four of these were positive for intimin-α, three carried H34, two carried H6, but all were of different serotypes. No strain hybridised with both Bfp probes.

Some aEPEC strains from humans and animals express adhesins that are homologous to the K88 fimbriae of enterotoxigenic *E. coli *[21,28]. To determine if the aEPEC strains in our collection carried similar sequences, we probed these strains for the *fae *gene of K88, but none of the aEPEC hybridised with this probe, even when tested at low stringency.

### Adherence to HEp-2 cells

After incubation for three hours with HEp-2 cells, 54 (81%) of 67 aEPEC strains were adherent: 24 strains adhered in an aggregative pattern, and two in the pattern termed "localised-like adherence", because it resembles BFP-mediated localised adherence, but the bacteria are more loosely associated with each other than BFP-bearing strains. Twenty-eight strains showed an indeterminate pattern of adherence described previously [20], in which bacteria adhere in a mixed pattern of diffuse and localised-like adherence. Thirteen strains did not adhere to HEp-2 cells after 3 hours. Extending the contact period of the bacteria with the HEp-2 cells from 3 to 6 hours allowed some strains that had not adhered after 3 hours to adhere in an indeterminate pattern, but the pattern of strains that were adherent after 3 hours did not change.

Five of the six strains that hybridised with the BfpA probe were non-adherent after three hours, whereas five of the six BfpB-positive strains showed aggregative adherence and one showed localised-like adherence. Adherence to HEp-2 cells was not associated with a positive PCR for either Lpf or Efa.

### Association of specific virulence determinants with clinical presentation

The 67 aEPEC strains we investigated by PCR, DNA hybridisation and for adherence to HEp-2 cells originated from individuals with different clinical presentations (Table 2). Fifty-seven isolates were obtained from patients with diarrhoea, and ten were from asymptomatic individuals. Eleven isolates were from children with persistent diarrhoea (i.e., diarrhoea lasting more than 14 days), and 12 were from children with diarrhoea less than 14 days in duration. Thirty-four strains were from patients in whom the duration of diarrhoea was not known. To determine if any of the putative accessory virulence determinants of aEPEC that were sought in this study were associated with a particular clinical presentation, we compared the frequency of these determinants in isolates from patients with and without diarrhoea, and those known to have acute or persistent diarrhoea. The results showed that the frequency of the factors investigated did not differ significantly between the groups under comparison (P > 0.1, Fisher's exact test, two-tailed).

**Table 2:** Frequency of putative virulence-associated determinants of atypical EPEC strains in study subjects with different clinical presentations.

	No. of strains positive for:^a^					
	
Clinical presentation	BfpA	BfpB	Cdt	Efa1	Lpf_O113_	NleB1
						
All diarrhoea (n = 57)	5	6	7	7	13	18
No diarrhoea (n = 10)	1	0	0	1	0	2
Acute diarrhoea (n = 12)	1	4	4	2	3	3
Persistent diarrhoea (n = 11)	2	0	1	0	1	4

^a ^Only determinants that were present in more than three isolates overall were included in this analysis.

## Discussion

The classification of diarrhoeagenic strains of *E. coli *into pathotypes has led to considerable improvement in our understanding of the epidemiology, pathogenesis and clinical presentation of infections with these bacteria, and has spawned novel strategies to diagnose and prevent these infections [29,30]. Each pathotype of diarrhoeagenic *E. coli *carries a distinctive suite of virulence determinants, almost all of which show evidence of having been acquired on mobile genetic elements, such as plasmids, transposons, bacteriophages and pathogenicity islands. Interestingly, apart from their shared virulence determinants, strains of each pathotype often differ from each other in terms of serotype, biotype, phage type, and even with regard to the nature of the specific virulence determinants they carry, e.g., Shiga toxin-1 or Shiga toxin-2 in EHEC, and heat-labile or heat-stable enterotoxin in enterotoxigenic *E. coli *[30,31].

It was not surprising, therefore, that the clinical isolates of aEPEC we examined in this study were heterogeneous in every way we investigated them, including by using MLST to examine their phylogenetic relatedness. This analysis confirmed that some strains are closely related to tEPEC, while others are more like EHEC [32]. Indeed, one of the aims of this study was to determine if aEPEC obtained from patients with diarrhoea are derived from tEPEC that have lost pEAF [12], or LEE-positive STEC strains that have been cured of the Stx-encoding bacteriophage [17]. Phylogenetic analysis revealed that 3 aEPEC strains obtained from 75 humans in Australia or New Zealand belonged to EHEC clades, and 11 belonged to clades that contain tEPEC. None of these 14 isolates belonged to serotypes of highly virulent or epidemic EHEC or EPEC and none carried the gene for EHEC-haemolysin [14,20,33], suggesting that they did not recently arise from EHEC strains. On the other hand, it was not surprising that three aEPEC strains, which were clustered together with EHEC O157:H7, were serotype O55:H7, given the evidence that the latter appears to be the progenitor of EHEC O157:H7 [34].

Most of the strains we investigated (61 of 75) either belonged to distinctive aEPEC clades or could not be classified, indicating further that they did not arise from EHEC or tEPEC. Even those strains which clustered with EPEC or EHEC generally were of serotypes that are not common amongst tEPEC or STEC strains that are associated with infection of humans. Our finding that each bacterial isolate within each distinctive aEPEC clade generally carried the same intimin type mirrors observations made with tEPEC [35] and provides further evidence that *E. coli *acquired the LEE pathogenicity island on a number of separate occasions.

aEPEC in different clades did not differ from one another in terms of their association with acute or persistent diarrhoea. This conclusion is in keeping with our somewhat unexpected finding that REPEC strains E22 and 83/39, which carry closely related virulence determinants, and are proven pathogens of infant rabbits in which they cause a similar illness, clustered with EPEC and EHEC, respectively.

Our search for virulence determinants in clinical isolates of aEPEC revealed that a minority of strains carried homologues of DNA sequences that encode known adhesins or other virulence-associated determinants of pathogenic *E. coli*. Overall, six strains each hybridised with DNA probes for BfpA and BfpB, respectively, and PCR analysis gave positive results for Lpf (13 strains), Iha (3 strains), AF/R1 (2 strains), Afa (1 strain), or AggA (1 strain). To our knowledge, this is the first time that AF/R1 has been identified in any *E. coli *other than the prototypical REPEC strain, RDEC-1 [36], but we have not determined if this gene or any of the other putative virulence genes in the probe- or PCR-positive aEPEC strains we investigated is expressed by the strain that carries it. Moreover, no strain was positive in the PCR for *ldaH*, which is the only known specific adhesin of aEPEC identified so far [28].

In this study we were unable to confirm previous reports that *nleB *or *efa1*, which are key components of a genomic island of EPEC and virulent STEC [37], are markers of symptomatic infection with aEPEC [38], largely because these determinants were present in so few strains (present in only 20 and 8 of 67 strains, respectively). We also did not find any association between the presence of any genes for particular virulence determinants and the clinical presentation of patients in terms of the presence or duration of diarrhoea, but the small number of probe- or PCR-positive strains made the finding of statistically significant associations unlikely.

All of the aEPEC strains we investigated in this study expressed functional Type I pili. Although these pili are widespread amongst all varieties of *E. coli*, including non-pathogens, evidence is accumulating that these pili, which are well established virulence determinants of uropathogenic and systemically invasive *E. coli *[39,40], may also contribute to the virulence of EPEC and enteroaggregative *E. coli*, particularly with respect to biofilm formation [41,42]. Type I pili are also an essential virulence determinant of adherent-invasive *E. coli *[43]. In addition, overexpression of Type I pili by a BFP-mutant of tEPEC was able to compensate for the absence of BFP and allowed bacteria to adhere to cultured epithelial cells in vitro [44]. Whether Type I pili contribute to the virulence of aEPEC, however, remains to be determined.

## Conclusion

Our findings show that aEPEC are highly heterogeneous in terms of serotype, intimin type, multilocus sequence type, pattern of adherence to HEp-2 cells, and their carriage of known virulence genes (apart from those encoded by the LEE). Although we did not identify a common type of adhesive fimbria in aEPEC that is functionally equivalent to BFP, we cannot rule out that one exists. Indeed, the fact that all tEPEC strains express BFP despite their phylogenetic heterogeneity supports the case for continued efforts to identify specific adhesins of aEPEC.

## Methods

### Bacteria

For the purposes of this study, aEPEC were defined as strains of *E. coli *that were positive by PCR for the *eae *gene, but negative by PCR for the genes for BfpA and Shiga toxins 1 and 2, using the PCR primers and conditions described previously [14]. Sixty-seven of the aEPEC strains investigated in this study were isolated in our laboratory during the course of four separate studies: a Melbourne water-quality study (21 strains with a prefix of W [20], a study examining the clinical features of children infected with aEPEC at the Royal Children's Hospital, Melbourne, Australia (28 strains with a prefix of R [14]), a study of the prevalence of STEC in Melbourne (3 strains, prefixed ESA), and a study of acute diarrhoea in children in the Northern Territory, Australia (15 strains, prefixed NT [33]). The remaining clinical aEPEC isolates were E128012, from a case of sporadic infant diarrhoea in Bangladesh [12], F41 (Denmark [45]), E65/56 and D5301 (England [46-48]), all of which are archetypal aEPEC strains [49]. We also tested 8 clinical aEPEC strains from New Zealand (kindly supplied by Jenny Bennett, ESR Ltd., Porirua, New Zealand) and eight aEPEC strains isolated from symptomatic cattle in Australia [18] (kindly supplied by Dr Steven Djordjevic, Elizabeth Macarthur Agricultural Institute, Camden, NSW, Australia).

Reference strains of *E. coli *included in the phylogenetic analysis of the aEPEC strains were: tEPEC (*eae*+ *bfpA*+) strains, E2348/69, E990, Stoke W and C771 [12,49]; REPEC strains, E22 [50], 83/39, 84/110-1 [51], and an STEC O157:H7 strain, EDL933, which is LEE-positive and classified as enterohemorrhagic *E. coli *(EHEC) [52]. *E. coli *strains used as controls for PCR included enteroaggregative *E. coli *strain 17-2 [53]; STEC strains, EH41 [54], and EH52 (this study); enterotoxigenic *E. coli *strain K88 and *E. coli *K12-K99+ (courtesy of Professor Peter Reeves, University of Sydney, Sydney, NSW, Australia); REPEC strains, B10 [55], 83/39 and RDEC-1 [56], and uropathogenic *E. coli *strain J96 [57]. Adherent-invasive *E. coli *strain LF82, which was isolated from a chronic ileal lesion of a patient with Crohn's disease, and 52D11 (an isogenic *fimA *mutant of LF82) [43] were kindly supplied by Dr Arlette Darfeuille-Michaud, Université d'Auvergne, Clermont-Ferrand, France, and used as controls to test for mannose-sensitive haemagglutination. Unless otherwise specified, bacteria were routinely subcultured on horse blood agar or Luria-Bertani agar (BD Difco, Franklin Lakes, NJ) at 37°C.

### Preparation of DNA

Genomic DNA was isolated from *E. coli *using hexadecyltrimethylammonium bromide (CTAB) as described in Ausubel et al. [58], and was used as the template for all experiments requiring DNA.

### Multi-locus sequence typing (MLST)

Eighty-three test strains isolated from humans or cattle in Australia and New Zealand, together with four archetypal aEPEC and eight A/E *E. coli *control strains were subjected to MLST analysis using the methods described on the EcMLST website http://www.shigatox.net/mlst. Briefly, seven housekeeping genes (*aspC*, *clpX*, *fadD*, *icdA*, *lysP*, *mdh *and *uidA*) were amplified with AmpliTaq Gold in 50 μl reaction volumes. PCR products (5 μl) were electrophoresed on 1% agarose gels to check the size and yield. The remaining 45 μl was purified using the QIAquick PCR Purification Kit (Qiagen, Valencia, CA) and eluted in 20 μl elution buffer. Both strands of each gene were sequenced using ABI PRISM BigDye Terminator (Applied Biosystems, Foster City, CA) according to the manufacturer's instructions. Sequences were checked and cropped to the required length using Sequencher 4.0 (Gene Codes, Ann Arbor, MI). Sequences were concatenated for phylogenetic analyses, and aligned with the ClustalW algorithm using the MegAlign module of the Lasergene software (DNASTAR Inc., Madison, WI). Neighbour-joining trees were constructed using the Kimura two-parameter model of nucleotide substitution with the MEGA3 software (Center for Evolutionary Functional Genomics, Tempe, AZ) [59]. The inferred phylogenies were each tested with 500 bootstrap replications.

### Accession numbers

The sequences of the *aspC, clpX*, *fadD*, *icdA*, *lysP*, *mdh *and *uidA *genes used for the MLST analysis have been deposited in the GenBank data base under accession numbers GQ130379 to GQ131022.

### Intimin typing

The *eae *gene was subtyped by using the restriction fragment length polymorphism assay described by Ramachandran et al. [60]. This method permits detection of the following intimin types: α (alpha), β (beta), β2, γ (gamma), ε (epsilon), ζ (zeta), θ (theta), ι (iota), κ (kappa), λ (lambda), ν (nu), ξ (xi), o (omicron), ρ (rho), and σ (sigma).

### Detection of genes for adhesins and other virulence factors by using PCR

PCR amplifications were performed in a GeneAmp PCR System 9700 thermal cycler (Applied Biosystems) or an iCycler (Bio-Rad Laboratories, Hercules, CA) with AmpliTaq Gold polymerase (Applied Biosystems) in a reaction volume of 20 μl. The genes, primers, amplicon size and PCR conditions used for these studies are listed in the additional file (see Additional file 1). The test strains for these analyses, and those described below, were the 67 aEPEC strains obtained from humans in Australia. The following *E. coli *strains were used as positive controls: E2348/69 (*bfpA*), 83/39 (*efa1*, *ralG*), EDL933 (*iha, nleB1*), EH41 (*saa*, *lpfD*_O113_); K88 (*fae *operon), K12-K99+ (*fan *operon), 17-2 (*aggA*); J96 (*fimH*, *papA*, *sfa/focDE*, *focG*), EH52 (*afaC*), RDEC-1 (*afr1*), B10 (*afr2*), and E990 (*cdt*). PCR products were electrophoresed on 1–1.5% Tris-acetate-EDTA agarose gels and stained with ethidium bromide before visualisation on a UV transilluminator.

### DNA Hybridisation

Genomic DNA was spotted onto Magna Nylon Transfer Membranes (GE Osmonics, Trevose, PA) and denatured and neutralised according to the "DIG System User's Guide for Filter Hybridisation" (Roche, Mannheim, Germany). Transferred DNA was UV-crosslinked using a Spectrolinker XL-1000 UV crosslinker (Spectronics Corp., Westbury, NY). Digoxigenin-labelled DNA probes were prepared by PCR (Roche) using primers to detect *bfpA *(Table 1); primers MP-bfpB-F (GATAAAACTGATACTGGGCAGC) and MP-bfpB-R (AGTGACTGTTCGGGAAGCAC) to detect *bfpB *[61]; and primers faeEF (ATGCGCCGGGTGATATCA) and faeER (TTATTTCTGCTCTGCGGT) to detect *faeE*. EPEC E2348/69 was used as template for the *bfpA *and *bfpB *probes and enterotoxigenic *E. coli *strain K88 was used as template for the *faeE *probe. These strains were also included as positive controls on the appropriate membranes. Before use, probes were sequenced using ABI PRISM Big Dye Terminator as described above. Sequencing reactions were purified using MgSO_4 _and submitted to the Australian Genome Research Facility (Parkville, Vic, Australia). Hybridisation was performed at low stringency (hybridisation at 50°C followed by washing in 2× SSC at room temperature), medium stringency (hybridisation at 59°C, washing in 0.1× SSC at 59°C), and high stringency (hybridisation at 68°C, washing in 0.1× SSC at 68°C), and detected by using chemiluminescence as recommended by the manufacturer. Bacteria were considered probe-positive if the intensity of the spot was similar to that of the positive control.

### Bacterial adherence to HEp-2 cells

The Center for Vaccine Development method was used to determine the pattern of bacterial adherence to HEp-2 epithelial cells [62]. The criteria used to assign bacterial adherence to a particular pattern have been described previously [20].

### Type I pili production

The expression of Type I pili was determined by investigating bacteria for mannose-sensitive haemagglutination of guinea pig erythrocytes. For these assays, bacteria were grown in Brain Heart Infusion broth (Oxoid Ltd., Basingstoke, England) or Antibiotic Medium No. 3 (Penassay broth, PAB; Oxoid Ltd.) at 37°C without shaking, and tested for haemagglutination using the method described by Iida et al. [63]. *E. coli *strains, LF82 and 52D11, were used as positive and negative controls, respectively. Strains that were haemagglutination-negative were retested after passage through Brain Heart Infusion broth to enhance the expression of Type I pili.

## Abbreviations used

A/E: attacing-effacing; aEPEC: atypical EPEC; BFP: bundle-forming pili; BHIB: brain heart infusion broth; Cdt: cytolethal distending toxin; EHEC: enterohaemorrhagic *Escherichia coli*; EPEC: enteropathogenic *E. coli*; LEE: locus for enterocyte effacement; Lpf: long polar fimbriae; MLST: multi-locus sequence typing; pEAF: EPEC adherence factor plasmid; REPEC: rabbit-specific EPEC; STEC: Shiga toxin-producing *E. coli*; tEPEC: typical EPEC.

## Authors' contributions

SMT and MT contributed to the design of the study, performed the PCR and assays and contributed to the preparation of the manuscript. KA, AB and VBW performed the hybridisation, haemagglutination and tissue culture assays and contributed to the preparation of the manuscript. WQ and TSW interpreted the raw MSLT data and contributed to the preparation of the manuscript. RMRB conceived and designed the study and oversaw the preparation of the manuscript. All authors read and approved the final manuscript.

## Supplementary Material

Additional file 1 **PCR primers and conditions used in this study, and sizes of PCR amplicons**. Click here for file
