# Supplementary material for: Characterisation of atypical enteropathogenic E. coli strains of clinical origin
Source: BMC Microbiol. 2009 Jun 3;9:117. doi: 10.1186/1471-2180-9-117 (PMC2700815; doi:10.1186/1471-2180-9-117)
Supplement: Additional file 1 — PCR primers and conditions used in this study, and sizes of PCR amplicons. [file 1471-2180-9-117-S1.pdf]

Additional file 1: PCR primers and conditions used in this study, and sizes of PCR amplicons.

Target	Forward primer	Reverse primer	PCR conditions[a]	Amplicon (bp)	Ref
afaC	CGGCTTTTCTGCTGAACTGGCAGGC	CCGTCAGCCCCCACGGCAGACC	94ºC 30 s, 72ºC 1 min	672	[1]
afr1	TACCGTTACTGCGAAGACCT	CGTGCTGTTAATCGCCACTA	94ºC 45 s, 60ºC 45 s, 72ºC 45 s	280	[2]
afr2	AAGTTAGGGGACGCCATTAC	CCAGGACTTATTCTGACCAG	94ºC 45 s, 57ºC 45 s, 72ºC 45 s	518	[2]
aggA	ATGCATTACTTTGGGTTTAG	TCAACCTTGACACTTGCC	94ºC 30 s, 50ºC 30 s, 72ºC 30 s	414	[3]
bfpA	ATTGAATCTGCAATGGTGC	ATAGCAGTCGATTTAGCAGCC	95ºC 30 s, 58ºC 30 s, 72ºC 30 s	461	[3]
cdt	GAAARTAAATGGAAYAYAMATGTCCG	AATCWCCWRSAATCATCCAGTTA	94ºC 1 min, 55ºC 1 min, 72ºC 1 min	466	[4]
efa1	AAGGTGTTACAGAGATTA	TGAGGCGGCAGGATAGTT	94ºC 30 s, 55ºC 30 s, 72ºC 30 s	268	[5]
fimH	TGCAGAACGGATAAGCCGTGG	GCAGTCACCTGCCCTCCGGTA	94ºC 30 s, 60ºC 30 s, 72ºC 30 s	508	[6]
focG	CAGCACAGGCAGTGGATACGA	GAATGTCGCCTGCCCATTGCT	94ºC 30 s, 63ºC 30 s, 72ºC 30 s	360	[6]
iha	CAGTTCAGTTTCGCATTCACC	GTATGGCTCTGATGCGATG	94ºC 1 min, 52ºC 1 min, 72ºC 1.5 min	1305	[7]
K88	ATCGGTGGTAGTATCACTGC	AGAACCTGCGACGTCAACAAGA	94ºC 30 s, 60ºC 30 s, 72ºC 45 s	595	[8]
K99	TGCGACTACCAATGCTTCTG	TATCCACCATTAGACGGAGC	94ºC 30 s, 60ºC 30 s, 72ºC 30 s	450	[8]
ldaH	ATGAATAACATGAAAGTAATTCG	TCATTGCACAGTAACCGTAAC	95ºC 30 s, 58ºC 30 s, 72ºC 1 min	807	This study
$lpfD_{O113}$	GAACTGTAGATGGGTAC	AGCAGGCATAACGCAAG	94ºC 1 min, 48ºC 50 s, 72ºC 1 min	798	[9]
nleB1	GGTGTGCTGGTAGATGGA	CAGGGTATGATTCTTGTTTATG	94ºC 30 s, 53ºC 30 s, 72ºC 30 s	175	[9]
papA	GTTATTGCCGGTGCGGTA	AATTCGCAACTGCTGAGA	94ºC 30 s, 55ºC 30 s, 72ºC 30 s	530	[10]
ralG	CGTCACCTCTGGAGTGGC	GGAAGCACCGATGCCAGC	94ºC 30 s, 60ºC 30 s, 72ºC 30 s	267	This study
saa	CGTGATGAACAGGCTATTGC	ATGGACATGCCTGTGGCAAC	94ºC 30 s, 60ºC 30 s, 72ºC 30 s	119	[11]
sfa/focDE	CTCCGGAGAACTGGGTGCATCTTAC	CGGAGGAGTAATTACAAACCTGGCA	94ºC 30 s, 63ºC 30 s, 72ºC 30 s	410	[12]

[a] 30 cycles. Plus an initial denaturation of 95ºC for 10 min and final extension at 72ºC for 5 min.

[b] M = AC, R = AG, S = GC, W = AT, Y = CT

References

1. Le Bouguénec C, Lalioui L, du Merle L, Jouve M, Courcoux P, Bouzari S, Selvarangan R, Nowicki BJ, Germani Y, Andremont A, Gounon P, Garcia MI.: **Characterization of AfaE adhesins produced by extraintestinal and intestinal human *Escherichia coli* isolates: PCR assays for detection of Afa adhesins that do or do not recognize Dr blood group antigens**. *J Clin Microbiol* 2001, **39:**1738-1745.
2. Dow MA, Toth I, Alexa P, Davies M, Malik A, Oswald E, Nagy B: **Predominance of *afr2* and *ral* fimbrial genes related to those encoding the K88 and CS31A fimbrial adhesins in enteropathogenic *Escherichia coli* isolates from rabbits with postweaning diarrhea in Central Europe**. *J Clin Microbiol* 2005, **43:**1366-1371.
3. Robins-Browne RM, Bordun A-M, Tauschek M, Bennett-Wood V, Russell J, Oppedisano F, Lister NA, Bettelheim KA, Fairley CK, Sinclair MI, Hellard ME: **Atypical enteropathogenic *Escherichia coli*: a leading cause of community-acquired gastroenteritis in Melbourne, Australia**. *Emerg Infect Dis* 2004, **10:**1797-1805.
4. Toth I, Herault F, Beutin L, Oswald E: **Production of cytolethal distending toxins by pathogenic *Escherichia coli* strains isolated from human and animal sources: establishment of the existence of a new cdt variant (Type IV)**. *J Clin Microbiol* 2003, **41:**4285-4291.
5. Nicholls L, Grant TH, Robins-Browne RM: **Identification of a novel locus that is required for in vitro adhesion of a clinical isolate of enterohaemorrhagic *Escherichia coli* to epithelial cells.** *Mol Microbiol* 2000, **35:**275-288.
6. Johnson JR, Stell AL: **Extended virulence genotypes of *Escherichia coli* strains from patients with urosepsis in relation to phylogeny and host compromise**. *J Infect Dis* 2000, **181:**261-272.
7. Toma C, Martinez EE, Song T, Miliwebsky E, Chinen I, Iyoda S, Iwanaga M, Rivas M: **Distribution of putative adhesins in different seropathotypes of Shiga toxin-producing *Escherichia coli*.** *J Clin Microbiol* 2004, **42:**4937-4946.
8. Korczak B, Frey J, Schrenzel J, Pluschke G, Pfister R, Ehricht R, Kuhnert P: **Use of diagnostic microarrays for determination of virulence gene patterns of *Escherichia coli* K1, a major cause of neonatal meningitis**. *J Clin Microbiol* 2005, **43:**1024-1031.
9. Doughty S, Sloan J, Bennett-Wood V, Robertson M, Robins-Browne RM, Hartland EL: **Identification of a novel fimbrial gene cluster related to long polar fimbriae in locus of enterocyte effacement-negative strains of enterohemorrhagic *Escherichia coli*.** *Infect Immun* 2002, **70:**6761-6769.
10. Kuhnert P, Hacker J, Muhldorfer I, Burnens AP, Nicolet J, Frey J: **Detection system for *Escherichia coli*-specific virulence genes: absence of virulence determinants in B and C strains**. *Appl Environ Microbiol* 1997, **63:**703-709.
11. Paton AW, Paton JC: **Direct detection and characterization of Shiga toxigenic *Escherichia coli* by multiplex PCR for *stx1*, *stx2*, *eae*, *ehxA*, and *saa*.** *J Clin Microbiol* 2002, **40:**271-274.
12. Le Bouguénec C, Archambaud M, Labigne A: **Rapid and specific detection of the *pap*, *afa*, and *sfa* adhesin-encoding operons in uropathogenic *Escherichia coli* strains by polymerase chain reaction**. *J Clin Microbiol* 1992, **30:**1189-1193.
